# Psychometric properties of the Sexual Excitation/Sexual Inhibition Inventory for Women and Men (SESII-W/M) and the Sexual Excitation Scales/Sexual Inhibition Scales short form (SIS/SES-SF) in a population-based sample in Germany

**DOI:** 10.1371/journal.pone.0193080

**Published:** 2018-03-12

**Authors:** Julia Velten, Saskia Scholten, Jürgen Margraf

**Affiliations:** Mental Health Research and Treatment Center, Ruhr-Universität Bochum, Bochum, Germany; Fundación Universitaria Konrad Lorenz, COLOMBIA

## Abstract

The Sexual Excitation Sexual/Inhibition Inventory for Women and Men (SESII-W/M) and the Sexual Excitation Scales/Sexual Inhibition Scales short form (SIS/SES-SF) are two self-report questionnaires for assessing sexual excitation (SE) and sexual inhibition (SI). According to the dual control model of sexual response, SE and SI differ between individuals and influence the occurrence of sexual arousal in given situations. Extreme levels of SE and SI are postulated to be associated with sexual difficulties or risky sexual behaviors. The present study was designed to assess the psychometric properties of the German versions of both questionnaires utilizing a large population-based sample of 2,708 participants (*M*_age_ = 51.19, *SD* = 14.03). Overall, psychometric evaluation of the two instruments yielded good convergent and discriminant validity and mediocre to good internal consistency. The original 30-item version of the SESII-W/M did not show a sufficient model fit. For a 24-item version of the SESII-W/M partial strong measurement invariance across gender, and strong measurement invariance across relationship status, age, and educational levels were established. The original structure (14 items, 3 factors) of the SIS/SES-SF was not replicated. However, a 4-factor model including 13 items showed a good model fit and strong measurement invariance across the before-mentioned participant groups. For both questionnaires, partial strong measurement invariance with the original American versions of the scales was found. As some factors showed unsatisfactory internal consistency and the factor structure of the original scales could not be replicated, scores on several SE- and SI-factors should be interpreted with caution. However, most analyses indicated sufficient psychometric quality of the German SESII-W/M and SIS/SES-SF and their use can be recommended in German-speaking samples. More research with diverse samples (i.e., different sexual orientations, individuals with sexual difficulties) is needed to ensure the replicability of the factor solutions presented in this study.

## Introduction

The dual control model of sexual response offers a theoretical framework to systematically research human sexuality and to explain individual differences in sexual behaviors, interests, and responses [1,2]. According to this model, an individual’s sexual motivation is based on two relatively independent propensities, sexual excitation (SE) and sexual inhibition (SI) that vary from person to person. Assuming a normal distribution of the two propensities, most levels of SE and SI are expected to lead to relatively functional and adaptive sexual behaviors. Extreme levels of SE and SI, however, are associated with increased risks for problematic or maladaptive sexual behaviors [1,3]. There is growing evidence that high levels of SI and low levels of SE are associated with increased vulnerability for sexual dysfunctions [3–6]. Additionally, high SE and low SI increase the likelihood of out-of-control sexual behaviors, like excessive use of pornography, and risky sexual behaviors, such as unprotected intercourse [7–10].

### Assessment of sexual excitation and sexual inhibition

To allow systematic testing of the dual control model’s propositions, several questionnaires have been developed. The first questionnaire created to assess SE and SI was the 45-item Sexual Inhibition/Sexual Excitation Scales (SIS/SES) [11]. This scale has a 3-dimensional factor structure with one sexual excitation scale (SES) and two sexual inhibition scales (SIS1 and SIS2). The SES’ items describe stimuli or situations that are potentially sexually arousing, like seeing an attractive person or watching an erotic video. SIS1 assesses inhibition due to the threat of performance failure. The items describe situations in which distracting thoughts or pressure to perform lead to the loss of an erection or reduced arousal. SIS2 describes inhibition due to anticipated negative consequences of sexual encounters. The items include statements about loss of arousal or erection due to the fear of sexually transmitted infections or the risk of being caught during sexual activity. Most psychometric properties of the SIS/SES have been found satisfactory to good in men and women [11,12]; the factor structure, however, showed a better fit in male compared to female samples.

Investigating between-group differences in SE and SI (i.e., between men and women, heterosexual or homosexual individuals, younger or older persons) may help to explain group differences in sexual dysfunctions or sexual behaviors [13,14]. Gender comparisons using the SIS/SES indicated that men in general report significantly higher levels of SE, while women report higher SI [12]. These group-differences, however, can only be interpreted with caution as an important methodological requirement to allow such comparisons, namely measurement invariance, was not tested [15]. Measurement invariance implies that group-comparisons are valid because the respective scale measures the same underlying factors in all groups under investigation. In other words, if an instrument is measurement invariant, observed scores do not depend on group membership [15]. This means that members of different groups who have the same score on a factor (e.g., the same level of SE) have on average the same observed scores (for more information on the different levels of invariance, please refer to the Data Analysis section of this paper).

In 2013, a 14-item short form of the SIS/SES, called SIS/SES-SF, was published including the same factor structure and a selection of items that were found measurement invariant across genders [16]. However, information about the model fit was not reported. Retest-reliability (*M*_days_ = 40) of the U.S. American SIS/SES-SF was mediocre to good (.61 < *r* < .75) [16]. Internal consistency was not reported. Convergent and discriminant validity of the SIS/SES-SF was evaluated by correlations with other questionnaires that measure proximal and distal constructs [16]. SIS1 and SIS2 were moderately negatively correlated with behavioral inhibition and SES showed positive correlations with behavioral activation [17]. SES showed positive, SIS1 and SIS2 showed negative correlations with sociosexual orientation (SOI) [18], whereby higher levels of SOI indicate a more casual attitude towards sex outside committed relationships [19]. None of the scales were significantly correlated with a measure of social desirability [20]. Using the SIS/SES-SF in a representative survey of the general population in Flanders, Belgium, SE and SI showed a close to normal distribution and, additionally, the proposed gender differences were replicated [14,21]. As measurement invariance of the Belgian version was not reported, it remains unclear if the requirements for such gender comparisons were met and whether the gender differences can be validly interpreted.

Despite the promising validity of the SIS/SES and its short form, it remained unclear whether the instruments sufficiently reflected aspects that are particularly relevant for sexual arousal or response in women. Using on a focus group approach [22], a 115-item-pool was developed. Based in these items, the 36-item Sexual Excitation/Sexual Inhibition Inventory for Women (SESII-W) [23] and the 30-item Sexual Excitation/Sexual Inhibition Inventory for Women and Men (SESII-W/M) [24] were developed. The latter included only items that were measurement invariant across genders. Retest-reliability of the SESII-W/M was acceptable with correlations ranging from *r* = .66 to *r* = .82, with a mean correlation of *r* = .76. Good construct validity was found with SE- and SI-scales showing significant correlations to related constructs in the expected directions. Scales related to SI showed positive, small to medium correlations with behavioral inhibition [24]. SE-scales correlated positively with aspects of behavioral activation. In addition, SI-scales correlated negatively, SE-scales positively with sexual sensation seeking [24] which describes the propensity to pursue new and risky sexual situations [25,26].

### Assessment of sexual excitation and sexual inhibition in German samples

To assess SE and SI in non-English-speaking countries, researchers have translated questionnaires into other European languages such as Spanish, Portuguese, Dutch, or Polish (for an overview see [3]). In addition, the SIS/SES was translated into five Asian languages [27]. German versions of the SIS/SES, SIS/SES-SF [28], and SESII-W [29] have been developed. The only published validation study, however, described the psychometric properties of the SESII-W in a sample of 2,200 women [29]. The German SESII-W showed sufficient test-retest-reliability, internal consistency, and construct validity to allow for an assessment of SE and SI in German-speaking women.

### The present study

The main goal of this study was to describe the psychometric properties of the German SESII-W/M and SIS/SES-SF, two questionnaires that measure SE and SI in both genders. Therefore, a large sample was recruited to be representative of the German residential population. While a translated version of the SIS/SES-SF was available [28], the SESII-W/M was translated by the authors following principles of good practice for patient-related outcome measures [30]. Research has shown that men compared to women show higher levels of SE and lower levels of SI [21,24]. In addition, several studies have identified age-related differences in both factors [21]. Even though the need for more diverse and representative samples has been acknowledged [29], most studies still report findings based on highly educated, young student samples. This may be especially problematic, as a population-based study suggested that both SE and SI are increased in more highly educated individuals [14]. Also, it can be hypothesized that some items or scales measuring SE and SI may work differently for single and partnered individuals. For instance, the impact of partner behaviors (i.e., doing chores) on sexual response or the relevance of trust and commitment for sexual arousal may be different depending on the availability of a steady relationship. As sexual desire often declines in long-term relationships [31], relationship status may significantly impact SE.

Using the complete sample, we expected the factor structure to resemble the original U.S. American versions of the questionnaires [16,24]. To clarify if SESII-W/M and SIS/SES-SF can be used with participant groups that may be differ from those commonly used for the development of such questionnaires (i.e., undergraduate students), we investigated whether the factor structure of both questionnaires fitted the data of different subsamples: Men vs. women, younger vs. older, single vs. partnered participants, and persons with and without university degree. By testing the appropriateness of the factor solution in different subsamples, we assessed whether the questionnaires scales work similarly in different participant groups. In addition, we investigated if the SESII-W/M and SIS/SES-SF were measurement invariant across the before-mentioned subgroups. In case that the scales could be applied free from bias across the subgroups, latent means of different subsamples were compared.

Internal consistency was investigated as a measure of reliability. Furthermore, we assessed construct validity and hypothesized moderate correlations between the scales of both questionnaires and scales that measure other sexuality-related attitudes or behaviors such as sexual function, sociosexual orientation or the lifetime number of sexual partners, and expected negligible to small correlations with scales that measure distal factors such as life satisfaction or symptoms of anxiety/depression.

## Method

### Participants

For this study, three subgroups completed a survey on sexuality and partnerships: Individuals who were single (*n* = 522), were in a committed relationship, but completed the survey without their partner (*n* = 780), and couples where both partners participated (*n* = 1928). Ninety-seven percent (*n* = 2,580) identified as mostly or exclusively heterosexual, 0.7% (*n* = 20) as bisexual, 1.2% (*n* = 34) as mostly or exclusively homosexual, and 0.8% (*n* = 21) as asexual. Table 1 gives an overview of the sample characteristics.

**Table 1:** Sample characteristics of the complete sample (N = 2.708).

	*M (SD)*
Age (Range: 18–90)	51.19 (14.03)
Duration of partnership/being single (in years; Range: 0–66)	21.83 (14.70)
Children (Range: 0–8)	1.57 (1.21)
	*n*^a^ (%)
Marital status	
Married/Civil union	1936 (71.5)
Unmarried	398 (14.7)
Divorced	226 (8.3)
Widowed	126 (4.7)
Household income per month in Euro	
< 2,000	605 (22.3)
2,000–3,000	651 (24.0)
3,000–4,000	524 (19.4)
> 4,000	716 (26.4)
Educational level	
No high-school degree	1154 (42.6)
High-School degree	1515 (55.9)
Occupation	
Full-time occupation	1239 (45.8)
Part-time occupation	475 (17.5)
Retired	590 (21.8)
Housewife/House husband	155 (5.7)

Note.

^a^ Numbers vary due to missing data.

### Procedure

Computer-assisted telephone interviews were conducted for screening purposes and to gather participants’ informed consent. The study aimed to recruit a representative sample of the adult population. To accomplish representativeness, the sample was drawn from the residential population aged 18 years and older that was accessible via landline or mobile phones. Landline telephone numbers were chosen based on regional stratification while mobile phone numbers were stratified by providers. A within household random-sampling technique was used to facilitate random selection of individuals and to minimize sampling bias. During the telephone screening, it was assessed whether the respective household member was in a steady relationship. If the person answered affirmatively, the interviewer asked if he or she would be willing to participate in a study on relationship factors and sexuality together with his or her partner. After receiving detailed information about the study, informed consent of both partners was obtained verbally. Participants were assured that they could withdraw their consent at any given point without negative consequences. Individuals without a steady partner were also eligible and received a modified version of the questionnaire. All participants could choose to participate via online or paper-pencil survey. Study information (e.g., content, duration, and voluntariness) was presented again on the first page of the survey. The study was conducted from September 2015 to January 2016. Of the 8,153 identified target persons, 3,467 individuals (42.5%) gave their informed consent to participate either for themselves or––in case that the target person was in a steady relationship––for themselves and a partner. Of these individuals, 2,684 (77.44%) participated online (*n* = 1,621) or in paper-form (*n* = 1,063). Several differences in sociodemographic variables such as age, *F*(1,2698) = 14.05, *p* < .001, *d* = 0.61, emerged between these two participant groups. As both SESII-W/M and SIS/SES-SF showed strong measurement invariance across online- and paper-participants, differences between assessment methods were not described in this study. Please contact the first author for more information. All procedures were carried out in accordance with the provisions of the World Medical Association Declaration of Helsinki (2013). The Ethics Committee of the Faculty of Psychology at the Ruhr-Universität Bochum approved the study.

### Measures

#### Sexual Excitation/Sexual Inhibition Inventory for Women and Men (SESII-W/M)

This self-report questionnaire assesses SE and SI with 30 items that are answered on a Likert-type rating scale from 1 (*strongly disagree*) to 4 (*strongly agree*). This instrument was translated by the authors following the forward-backward translation procedure described by Wild et al. [30]. This procedure included different harmonization steps as well as a cognitive debriefing phase in which individuals naïve to the measure gave feedback to the comprehensibility and understandability of the instrument. The SESII-W/M has demonstrated good test-retest reliability as well as construct validity [24]. The questionnaire consists of three scales for SE and SI, respectively. Arousability (SE) includes five items that describe sexual arousability by a variety of stimuli such as seeing an attractive person. Partner characteristics and behaviors (SE) includes five items and describes how easily one becomes aroused while observing a sexual partner who is interacting well with others or showing his/her talent. Setting (SE) consists of four items that describe arousal related to unusual sexual situations or sexual situations in which one can be seen or overheard by others. Inhibitory cognitions (SI) consists of eight items which refer to cognitions or emotions that inhibit sexual arousal such as worry about having an orgasm or concerns about being a good lover, and feeling shy or self-conscious during sex. Dyadic elements of the sexual interaction (SI) covers with three items one’s needs regarding a sexual partner’s behavior in order to get aroused. The last scale, relationship importance (SI) consists of five items and emphasizes the need for trust and commitment in order to get aroused.

#### Sexual Inhibition Scales/Sexual Excitation Scales short form (SIS/SES-SF)

This self-report questionnaire assesses SE and SI with 14 items that are answered on a Likert-type rating scale from 1 (*strongly disagree*) to 4 (*strongly agree*). As described earlier, psychometric properties of the original version are satisfactory [16]. The U.S. American questionnaire includes three factors, one related to SE and two related to SI. The SES factor includes six items and refers to how different intrapersonal (i.e., phantasies) or interpersonal (i.e., a sexual partner) stimuli may increase sexual arousal. SIS1 consists of four items that describe how worries or concerns about sexual function may reduce or inhibit sexual arousal. SIS2 also consists of four items and refers to sexual inhibition related to potential negative consequences of sexual interactions (i.e., sexually transmitted infections).

#### Sexual function

Two questionnaires were used to measure sexual function in women and men. The Female Sexual Function Index (FSFI) [32] was used to assess sexual function in women. The FSFI consists of 19 items in six subscales (i.e., desire, arousal, lubrication, orgasm, satisfaction, and pain) that are answered on a 1- to 5-point scale, with higher scores indicating better sexual function. Some questions include the additional answer category of 0, indicating no sexual activity during the last month. Subscales can be combined into one total score, ranging from 1.2 to 36 points, with a clinical cut-off of 26.55; women scoring below that cut-off are deemed at risk for sexual dysfunction. The validation of the German FSFI yielded good psychometric properties [33]. In this study, internal consistency of the total scale was excellent with α = .97.

Men’s sexual function was assessed with the 15-item International Index of Erectile function (IIEF) [34]. Items are answered on a scale from 0 to 5, with higher scores indicating better sexual function. A total score ranging from 5 to 75 can be calculated. In a German validation study of the IIEF, a cut-off of 53 for the total scale was appropriate to identify men with erectile dysfunction [35]. Good psychometric properties of the IIEF have been found in various populations and language versions [35,36]. In this study, internal consistency was excellent with Cronbach’s α = .91.

#### Sociosexual orientation

The willingness to engage in uncommitted sexual relations was measured with the Revised Sociosexual Orientation Inventory (SOI-R) [19]. This revised scale consists of nine items which describe sociosexual behavior, attitude, and desire. Thus, it is a more differentiated measure than the original SOI [18]. Validity of the scale was demonstrated in two studies, as was internal consistency with a Cronbach’s α of .83/.84 in female samples [19]. The questionnaire and its revised version have been used in multiple studies on casual sexual behavior [37–39]. High scores on this measure are associated with more permissive attitudes toward engaging in uncommitted sexual relations, whereas low scores are associated with more negative attitudes [19].

#### Masturbation and number of sexual partners

The frequency of participants’ engagement in masturbation was assessed with the question “How often do you masturbate?” with a 6-point scale ranging from *never* to *5 times a week or more*. In this study, 22.1% reported not masturbating at all, 23.7% less than once a month, 24.5% once to thrice a month, 16.5% once or twice a week, 8.5% thrice to four times a week, and 4.7% reported masturbating more often than that. The number of lifetime sexual partners was assessed with the question “With how many different persons did you engage in sexual intercourse in your life?”. 2.8% indicated having had no sexual partner, 15.3% had one partner, 10.1% two, 9.5% three, 7.8% four, and 8.1% indicated five partners. More than 80% of participants reported ten or fewer sexual partners, more than 90% indicated 20 or fewer partners. Thirty participants (0.9%) indicated between 70 and 300 sexual partners.

#### Other measures

Life satisfaction and symptoms of anxiety and depression were included to assess the construct validity of the SESII-W/M and SIS/SES-SF. The 5-item Satisfaction with Life Scale (SWLS) [40] has good psychometric properties [41,42] and measures the judgmental component of personal wellbeing with five items rated on a scale ranging from 1 (*strongly disagree*) to 7 (*strongly agree*). Internal consistency of the SWLS was very good (α = .91) in the present sample. The Patient Health Questionnaire (PHQ-4) [43] is a short self-report scale that assesses symptoms of anxiety and depression over the last two weeks with 4-items ranging from 0 (*not at all*) to 3 (*nearly every day*). Internal consistency of this measure was satisfactory (α = .78) in the present sample.

### Data analyses

Data were analyzed using SPSS version 21.0 [44] and Mplus version 7.4 [45]. Across all variables under investigation, 4.1% of values were missing. Missing value analysis indicated data missing at random.

#### Factor structure

Confirmatory factor analyses (CFA) were conducted to assess if the factor structure of the German questionnaires resembled their American counterparts. To test the proposed models, two fit indices were evaluated: The comparative fit index (CFI) compares the hypothesized model’s χ2 with that resulting from the independence model. For an acceptable fit, CFI values above .90 are recommended; a good model fit requires values above .95 [46]. The Root Mean Square Error of Approximation (RMSEA) measures the difference between the reproduced covariance matrix and the population covariance matrix, with values less than .06 reflecting a small approximation error, indicating a good model fit, values between .08 and .10 a mediocre fit and values above 0.10 a poor model fit [47]. A χ2 statistic was reported for the sake of completeness as it is sensitive to large sample sizes, which leads to oversized rejection rates [48]. In cases where CFI and RMSEA indicated an unsatisfactory model-fit, modification indices were inspected to identify non-fitting items. These items were removed from the model until an acceptable model fit was achieved. If the deletion of items was not sufficient to improve model-fit, an exploratory factor analysis using Promax rotation was conducted to identify a factor structure that more adequately fit our data. This new factor structure was then used for CFA and subsequent measurement invariance testing. Parameters were estimated using robust weighted-least-squares (Weighted-Least-Squares Mean and Variance adjusted, WLSMV) [49,50]. WLSMV is recommended to estimate thresholds when fewer than five response categories are given [51].

#### Measurement invariance

To test whether the German SESII-W/M and SIS/SES-SF can be used comparably across different participant groups (i.e., gender, relationship status, age groups, and educational levels) measurement invariance was tested. This included a series of model comparisons. At each comparison step, equality constraints were added consecutively to the models [52]. In the baseline model (configural invariance), no equality constraints were made. This enabled an evaluation of whether factor structures were the same across groups. In the next step, factor loadings were constrained to be equal across participant groups. If this model fit the data and the fit was not substantially worse than the fit of the baseline model, weak or metric invariance was established. This means that the items measuring a factor are functioning equivalently or, in other words, that the unit of measurement is the same across groups and thus relationships among factors can be compared without bias. Subsequently, threshold invariance was tested constraining all thresholds to be equal across groups which is the adequate procedure for ordinal data. If threshold invariance is met, scalar or strong invariance can be assumed [53]. If strong measurement invariance could not be established, partial invariance was examined [52,54]. To test partial strong measurement invariance, first ill-specified items were identified by means of modification indices, then thresholds of these items were allowed to differ between groups. ΔCFI and ΔRMSEA were obtained calculating the difference between the CFI values, or RMSEA values respectively, for a more restricted model against a less restricted model. We considered the drop in the CFI-value and the increase of the RMSEA-value to evaluate the change of model fit. A change of ΔCFI ≥ .010 accompanied by a change of ΔRMSEA ≥ .015 indicated significant drop of model fit and hence non-invariance [55].

#### Latent mean differences

If at least partial strong measurement invariance was established, latent means of different groups were compared [48]. The comparisons were based on the model used to test strong invariance or partial strong invariance. However, as absolute values for latent means do not exist, only latent mean differences can be interpreted. Z-scores and p-values of the standardized model results are reported [56]. Cohen’s *d* was calculated as the effect size measure (small effect: *d* ≥ 0.20, medium effect: *d* ≥ 0.50, large effect: *d* ≥ 0.80) [56].

#### Descriptive analyses

Means, standard deviations, skewness, and kurtosis of the SESII-W/M and SIS/SES-SF were reported as descriptive variables. Absolute values larger than 2 for skewness or larger than 7 for kurtosis were considered as reference for substantial non-normality as is recommended for samples larger than 300 [57].

#### Reliability

Cronbach’s alpha indicated internal consistency of the scales and was considered acceptable above α > .70 [58].

#### Construct validity

Convergent and discriminant validity were assessed using bivariate correlations between the scales of the SESII-W/M, SIS/SES-SF, and related variables such as sexual function or sociosexual orientation as well as supposedly unrelated variables, like life satisfaction or symptoms of anxiety/depression. *R* ≥ .10 indicated a small, *r* ≥ .30 a medium, and *r* ≥ .50 a large effect size [59].

## Results

### Factor structure

#### SESII-WM

Using the complete sample, the overall fit of the original model—including 30 items and six factors—was tested. Fit indices suggested a rather poor model fit, χ^2^(390, *N* = 2,672) = 9225.019, *p* < .001, CFI = .767, RMSEA = .092. By eliminating six items (4, 18, 19, 21, 22, and 25) a satisfactory model fit was achieved, χ^2^(237, *N* = 2,671) = 2288.785, *p* < .001, CFI = .923, RMSEA = .057. An alternative approach for improving model fit would have been to allow items to load onto different factors; in this case, however, this was not sufficient to improve model fit. In addition, most ill-fitting items showed double loadings on factors related to both SE and SI. From a theoretical perspective, this would be undesirable as the dual control model suggests a relative independence of both propensities [1]. Thus, the authors decided to eliminate ill-fitting items, which offers a clean solution that other researchers can more easily apply to their datasets.

Table 2 shows the German and English wording of the items and the standardized factor loadings of the final model.

**Table 2:** Standardized factor loadings of each subscale of the 24-item version of the SESII-W/M in the complete sample.

Arousability (SE)	Original item	German translation	
9	I think about sex a lot when I am bored.	Ich denke viel an Sex, wenn mir langweilig ist.	.79
12	Just talking about sex is enough to put me in a sexual mood.	Allein über Sex zu reden genügt, um mich in sexuelle Stimmung zu versetzen.	.67
3	When I think about someone I find sexually attractive, I easily become sexually aroused.	Wenn ich an jemanden denke, den ich sexuell attraktiv finde, fällt es mir leicht, sexuell erregt zu werden.	.58
17	Sometimes I am so attracted to someone, I cannot stop myself from becoming sexually aroused.	Manchmal fühle ich mich zu jemandem so hingezogen, dass ich nicht verhindern kann, sexuell erregt zu werden.	.57
24	Just being physically close with a partner is enough to turn me on.	Einem Partner körperlich nah zu sein genügt bereits, um mich anzutörnen.	.55
**Partner characteristics and behaviors (SE)**			
23	If I see a partner interacting well with others, I am more easily sexually aroused.	Wenn ich sehe, dass ein Partner gut mit anderen auskommt, werde ich leichter sexuell erregt.	.61
10	I find it arousing when a partner does something nice for me.	Ich finde es erregend, wenn ein Partner etwas Nettes für mich tut.	.58
8	Someone doing something that shows he/she is intelligent turns me on.	Wenn jemand etwas tut, was seine Intelligenz zeigt, törnt mich das an.	.53
30	If a partner surprises me by doing chores, it sparks my sexual interest.	Wenn ein Partner mich überrascht indem er/sie den Haushalt macht, entfacht das mein sexuelles Interesse.	.45
**Setting (SE)**			
14 (rev.)	I find it harder to get sexually aroused if other people are nearby.	Ich finde es schwieriger sexuell erregt zu werden, wenn andere Menschen in der Nähe sind.	.81
5 (rev.)	If it is possible someone might see or hear us having sex, it is more difficult for me to get aroused.	Wenn die Möglichkeit besteht, dass uns jemand beim Sex sehen oder hören könnte, ist es schwieriger für mich, erregt zu werden.	.74
**Inhibitory cognitions (SI)**			
7	If I feel that I am expected to respond sexually, I have difficulty getting aroused.	Wenn ich spüre, dass eine sexuelle Reaktion von mir erwartet wird, habe ich Schwierigkeiten, erregt zu werden.	.75
15	If I think about whether I will have an orgasm, it is much harder for me to become aroused.	Wenn ich darüber nachdenke, ob ich zum Orgasmus komme, ist es für mich viel schwieriger, erregt zu werden.	.69
1	Sometimes I have so many worries that I am unable to get aroused.	Manchmal habe ich so viele Sorgen, dass ich nicht in der Lage bin, erregt zu werden.	.62
11	Sometimes I feel so ‘‘shy” or self-conscious during sex that I cannot become fully aroused.	Manchmal fühle ich mich beim Sex so schüchtern und unsicher, dass ich nicht vollständig erregt werden kann.	.61
29	If I am concerned about being a good lover, I am less likely to become aroused.	Wenn ich mir Sorgen darüber mache, ob ich ein/e gute/r Liebhaber/in bin, ist es unwahrscheinlicher, dass ich erregt werde.	.57
26	When I am having sex, I have to focus on my own sexual feelings in order to stay aroused.	Beim Sex muss ich mich auf meine eigenen sexuellen Gefühle konzentrieren, um erregt zu bleiben.	.54
**Dyadic elements of the sexual interaction (SI)**			
6	If I am uncertain how my partner feels about me, it is harder for me to get aroused.	Wenn ich unsicher bin, was ein Partner für mich empfindet, ist es schwieriger für mich, erregt zu werden.	.69
13	While having sex, it really decreases my arousal if my partner is not sensitive to the signals I am giving.	Beim Sex verringert es wirklich meine Erregung, wenn mein Partner nicht feinfühlig auf die Signale, die ich gebe, reagiert.	.62
20	If interferes with my arousal if there is not a balance of giving and receiving pleasure during sex..	Es beeinträchtigt meine Erregung, wenn es beim Sex kein Gleichgewicht zwischen Genussbereiten und -empfangen gibt	.53
**Relationship importance (SI)**			
28	I really need to trust a partner to become fully aroused.	Ich muss einem Partner wirklich vertrauen, um sexuell vollkommen erregt zu werden.	.72
27	If I think that a partner might hurt me emotionally, I put the brakes on sexually.	Wenn ich denke, dass ein Partner mich emotional verletzen könnte, blocke ich sexuell ab.	.64
16	It would be hard for me to become sexually aroused with someone who is involved with another person.	Es wäre schwierig für mich, bei jemandem sexuell erregt zu werden, der mit einer anderen Person eine Beziehung oder ein sexuelles Verhältnis hat.	.62
2	If I think that I am being used sexually it completely turns me off.	Wenn ich denke, dass ich sexuell benutzt werde, törnt mich das völlig ab.	.58

*Note*. The wording of the four items of the original scale that were not used in this factor solution is as follows:

Item 4: Seeing a partner doing something that shows his/her talent can make me very sexually aroused./Einen Partner dabei zu sehen, wie er/sie sein/ihr Talent unter Beweis stellt, kann mich sexuell sehr erregen.

Item 18: If I am very sexually attracted to someone, I don’t need to be in a relationship with that person to become sexually aroused./Wenn mich jemand sexuell stark anzieht, brauche ich nicht in einer Beziehung mit der Person sein, um sexuell erregt zu werden.

Item 19: Unless things are ‘‘just right” it is difficult for me to become sexually aroused./Es ist für mich schwierig, erregt zu werden, wenn nicht „alles richtig”ist.

Item 21: I get really turned on if I think I may get caught while having sex./Es törnt mich wirklich an, wenn ich daran denke, dass ich beim Sex erwischt werden könnte.

Item 22: If I am worried about taking too long to become aroused, this can interfere with my arousal./Wenn ich mir Sorgen darüber mache, dass ich zu lange brauche, um erregt zu werden, kann das meine Erregung beinträchtigen.

Item 25: Having sex in a different setting than usual is a real turn on for me./In einer anderen Umgebung als gewöhnlich Sex zu haben, törnt mich richtig an.

#### SIS/SES-SF

Overall fit of the original 3-factor model including all 14 items was insufficient as shown by the RMSEA index, χ^2^(74, *N* = 2,662) = 1880.594, *p* < .001, CFI = .909, RMSEA = .096. Eliminating items did not significantly improve the fit. Therefore, an exploratory factor analysis was conducted using the complete sample to identify whether another factor structure would better reflect our data. Principle component analysis with Promax rotation revealed four factors that explained 59.4% of variance. The first factor (SES1) that explained 25.3% of variance included Items 1, 3, 8, and 14 which were all items of the original SES scale. The second factor explained 18.4% of variance and included all items of the original SIS2 scale, namely Items 2, 5, 6, and 7. The third factor explained 8.2% of variance and included Items 4, 12, and 13; all part of the original SIS1. A fourth factor, explaining 7.4% of variance included items 9, 10, and 11. As Item 9 showed substantial double loadings with the third factor, it was excluded from further analyses. Therefore, the new factor consisted of two items (10 and 11) and was labeled SES2. Another CFA using the complete sample indicated a good fit of the 4-factor 13-item model, χ^2^(59, *N* = 2,661) = 664.026, *p* < .001, CFI = .969, RMSEA = .062. Using two separate random samples of our data set for EFA and CFA yielded a comparable model fit, χ^2^(59, *N* = 1,330) = 402.510, *p* < .001, CFI = .967, RMSEA = .066. Table 3 shows the wording of the English and German items and the standardized factor loadings of this model.

**Table 3:** Standardized factor loadings of each subscale of the 4-factor 13-item version of the SIS/SES-SF in the complete sample.

SES1	Original item	German translation	
14	When an attractive person flirts with me, I easily become sexually aroused.	Wenn eine attraktive Person mit mir flirtet, werde ich leicht sexuell erregt	.86
8	When I think of a very attractive person, I easily become sexually aroused.	Wenn ich an eine sehr attraktive Person denke, werde ich leicht erregt.	.82
1	When a sexually attractive stranger accidently touches me, I easily become aroused.	Wenn mich eine sexuell attraktive fremde Person zufällig berührt, werde ich leicht erregt.	.79
3	When I talk to someone on the telephone who has a sexy voice, I become sexually aroused.	Wenn ich mit einer Person am Telefon spreche, die eine sexy Stimme hat, werde ich sexuell erregt.	.65
**SES2**			
10	When I start phantasizing about sex, I quickly become sexually aroused.	Wenn ich anfange, über Sex zu fantasieren, werde ich schnell sexuell erregt.	.81
11	When I see others engaged in sexual activities, I feel like having sex myself.	Wenn ich andere bei sexuellen Handlungen sehe, möchte ich selbst Sex haben.	.78
**SIS1**			
12	When I have a distracting thought, I easily lose my erection/my arousal.	Wenn ich einen ablenkenden Gedanken habe, verliere ich leicht meine Erektion/ meine Erregung.	.76
4	I cannot get aroused unless I focus exclusively on sexual stimulation.	Ich kann nicht sexuell erregt werden, wenn ich mich nicht vollständig auf die sexuelle Stimulation konzentriere.	.66
13	If I am distracted by hearing music, television, or a conversation, I am unlikely to stay aroused.	Wenn ich durch das Hören von Musik, Fernsehen oder eine Unterhaltung abgelenkt bin, ist es unwahrscheinlich, dass ich erregt bleibe.	.64
**SIS2**			
7	If I can be seen by others while having sex, I am unlikely to stay aroused.	Wenn ich von anderen beim Sex beobachtet werden kann, ist es unwahrscheinlich, dass ich sexuell erregt bleibe.	.81
2	If I am having sex in a secluded, outdoor place and I think that someone is nearby, I am not likely to get very aroused.	Wenn ich Sex draußen, an einem abgeschiedenen Platz habe und denke, dass jemand in der Nähe ist, ist es unwahrscheinlich, dass ich sehr erregt werde	.70
5	If I am masturbating on my own and realize that someone is likely to come into the room at any moment, I will lose my erection/my sexual arousal.	Wenn ich mich allein selbst befriedige und mir bewusst wird, dass irgendjemand jederzeit in das Zimmer kommen kann, verliere ich meine Erektion / meine sexuelle Erregung.	.61
6	If I realize there is a risk of catching a sexually transmitted disease, I am unlikely to stay sexually aroused.	Wenn mir bewusst wird, dass das Risiko besteht, mich mit einer sexuell übertragbaren Krankheit anzustecken, ist es unwahrscheinlich, dass ich sexuell erregt bleibe.	.52

*Note*. The wording of the one item of the original scale that was not used in this factor solution is as follows:

Item 9: Once I have an erection/am sexually aroused, I want to start intercourse right away before I lose my erection/arousal./

Sobald ich eine Erektion habe/sexuell erregt bin, möchte ich sofort mit dem Geschlechtsverkehr beginnen, bevor ich meine Erektion/Erregung verliere.

### Measurement invariance

#### SESII-WM

In addition to the before-mentioned CFA using the complete German sample, eight single-group CFA were conducted (male vs. female, singles vs. partnered individuals, younger vs. older persons, and individuals with and without university degree). Furthermore, overall fit of the revised 24-item model within the U.S. validation sample of the original SESII-W/M [24] was assessed (see Table 4).

**Table 4:** Fit-Indices for single- and multi-group confirmatory factor analyses of the SESII-W/M (24-item version).

	*χ*^*2*^ *(df)*	CFI	RMSEA (90% CI)	ΔCFI	ΔRMSEA
*Single-Group-CFA*					
German sample	2288.785 (237)	.923	.057 (.055; .059)		
American sample	606.970 (237)	.949	.041 (.037; .045)		
Women	1510.220 (237)	.876	.063 (.060; .066)		
Men	1074.344 (237)	.930	.052 (.049; .055)		
Single individuals	483.351 (237)	.943	.045 (.040; .051)		
Partnered individuals	2167.162 (237)	.914	.061 (.059; .064)		
Younger participants (*M*_age_ = 39.77, *SD* = 9.02)	1309.921 (237)	.920	.059 (.056; .062)		
Older participants (*M*_age_ = 62.27, *SD* = 7.71)	1305.619 (237)	.912	.058 (.055; .061)		
Lower education	1405.424 (237)	.926	.055 (.052; .058)		
Higher education	1145.383 (237)	.915	.061 (.057; .065)		
*Multi-Group-CFA*					
*(1) German vs*. *American sample*					
Configural invariance	2810.400 (474)	.933	.052 (.051; .054)		
Weak/metric invariance	2903.366 (492)	.931	.052 (.050; .054)	|.002|	|.000|
Strong/threshold invariance	3439.722 (534)	.917	.055 (.053; .057)	|.014|	|.003|
Partial strong invariance (τ 3, τ 10 free)	3209.812 (528)	.923	.053 (.051; .055)	|.008|	|.001|
*(2) Women vs*. *Men*					
Configural invariance	2585.677 (474)	.904	.058 (.056; .060)		
Weak/metric invariance	2621.421 (492)	.903	.057 (.055; .059)	|.001|	|.001|
Strong/threshold invariance	3013.703 (534)	.888	.059 (.057; .061)	|.015|	|.002|
Partial strong (τ 1, τ 9 free)	2856.827 (528)	.894	.057 (.055; .060)	|.009|	|.000|
*(3) Singles vs*. *Partnered*					
Configural invariance	2503.367 (474)	.920	.057 (.054; .059)		
Weak/metric invariance	2487.009 (492)	.921	.055 (.053; .057)	|.001|	|.002|
Strong/threshold invariance	2621.636 (534)	.918	.054 (.052; .056)	|.002|	|.001|
*(4) Younger vs*. *older participants*					
Configural invariance	2621.373 (474)	.916	.058 (.056; .061)		
Weak/metric invariance	2650.333 (492)	.915	.057 (.057; .060)	|.000|	|.001|
Strong/threshold invariance	2833.176 (534)	.910	.057 (.055; .059)	|.005|	|.000|
*(5) Lower vs*. *higher education*					
Configural invariance	2548.596 (474)	.921	.057 (.055; .059)		
Weak/metric invariance	2544.869 (492)	.922	.056 (.054; .058)	|.001|	|.001|
Strong/threshold invariance	2655.567 (534)	.920	.055 (.052; .057)	|.002|	|.001|

*Note*. All χ^*2*^ values are significant, *p* < 001.

The model fit was good or very good in most participant groups including the U.S. American sample. In female participants, however, RMSEA indicated a good model fit, while the CFI did not quite meet the cut-off of an acceptable fit. As indicated by the fit indices and a drop of model fit between models that was below a ΔCFI ≤ .010 and a ΔRMSEA ≤ .015, multi-group CFA showed that the model was threshold measurement invariant across single and partnered, older and younger participants, as well as individuals with and without university degree. For the country and gender comparisons, a ΔCFI > .010 suggested that strong or scalar invariance cannot be assumed. However, by allowing the thresholds of Item 3 and Item 10 to vary across countries and the thresholds of Item 5 to vary across genders partial strong invariance was achieved.

#### SIS/SES-SF

Table 5 shows the results of the measurement invariance analysis for this questionnaire.

**Table 5:** Fit-Indices for single- and multi-group confirmatory factor analyses of the SIS/SES-SF (13-item version).

	χ^*2*^ *(df)*	CFI	RMSEA (90% CI)	ΔCFI	ΔRMSEA
*Single-Group-CFA*					
German sample	664.026 (59)	.969	.062 (.058; .066)		
American sample	273.820 (59)	.977	.042 (.037; .048)		
Women	301.468 (59)	.971	.055 (.049; .061)		
Men	355.416 (59)	.964	.062 (.056; .068)		
Single individuals	271.948 (59)	.930	.085 (.075; .095)		
Partnered individuals	517.461 (59)	.972	.060 (.055; .065)		
Younger participants (*M*_age_ = 39.77, *SD* = 9.02)	315.139 (59)	.971	.057 (.051; .064)		
Older participants (*M*_age_ = 62.27, *SD* = 7.71)	379.901 (59)	.969	.064 (.058; .070)		
Lower education	438.579 (59)	.966	.063 (.057; .068)		
Higher education	314.053 (59)	.969	.065 (.058; .072)		
*Multi-Group-CFA*					
*(1) German vs*. *American sample*					
Configural invariance	920.562 (118)	.973	.054 (.051; .057)		
Weak/metric invariance	1008.817 (127)	.970	.054 (.051; .058)	|.003|	|.000|
Strong/threshold invariance	1405.119 (149)	.957	.060 (.057; .063)	|.013|	|.006|
Partial strong invariance (τ 5 free)	3209.812 (528)	.962	.057 (.054; .060)	|.009|	|.003|
*(2) Women vs*. *Men*					
Configural invariance	658.145 (118)	.967	.059 (.054; .063)		
Weak/metric invariance	690.137 (127)	.966	.058 (.054; .062)	|.003|	|.001|
Strong/threshold invariance	859.833 (149)	.964	.060 (.056; .064)	|.002|	|.002|
*(3) Singles vs*. *Partnered*					
Configural invariance	778.863 (118)	.966	.065 (.061; .069)		
Weak/metric invariance	752.896 (127)	.968	.061 (.057; .065)	|.002|	|.004|
Strong/threshold invariance	754.364 (149)	.969	.055 (.051; .059)	|.001|	|.006|
*(4) Younger vs*. *older participants*					
Configural invariance	697.483 (118)	.969	.061 (.057; .065)		
Weak/metric invariance	779.857 (127)	.966	.062 (.058; .066)	|.003|	|.001|
Strong/threshold invariance	913.958 (149)	.960	.062 (.058; .066)	|.006|	|.000|
*(5) Lower vs*. *higher education*					
Configural invariance	754.039 (118)	.967	.064 (.059; .068)		
Weak/metric invariance	735.234 (127)	.968	.060 (.056; .064)	|.001|	|.004|
Strong/threshold invariance	757.030 (149)	.968	.055 (.052; .059)	|.000|	|.005|

*Note*. All χ^*2*^ values are significant, *p* < 001.

Single-group CFA revealed that the 4-factor 13-item model fit the data of most participant groups well. The model fit for single individuals was, however, only acceptable as indicated by a RMSEA > .08. Multi-group CFA showed that the model was threshold measurement invariant across genders, partnership status, age, and educational levels. For the country comparison, a ΔCFI of .013 suggested that strong invariance cannot be assumed. By allowing the thresholds of Item 5 to vary across countries partial strong invariance was achieved.

### Latent mean comparisons

#### SESII-W/M

**Fig 1:**
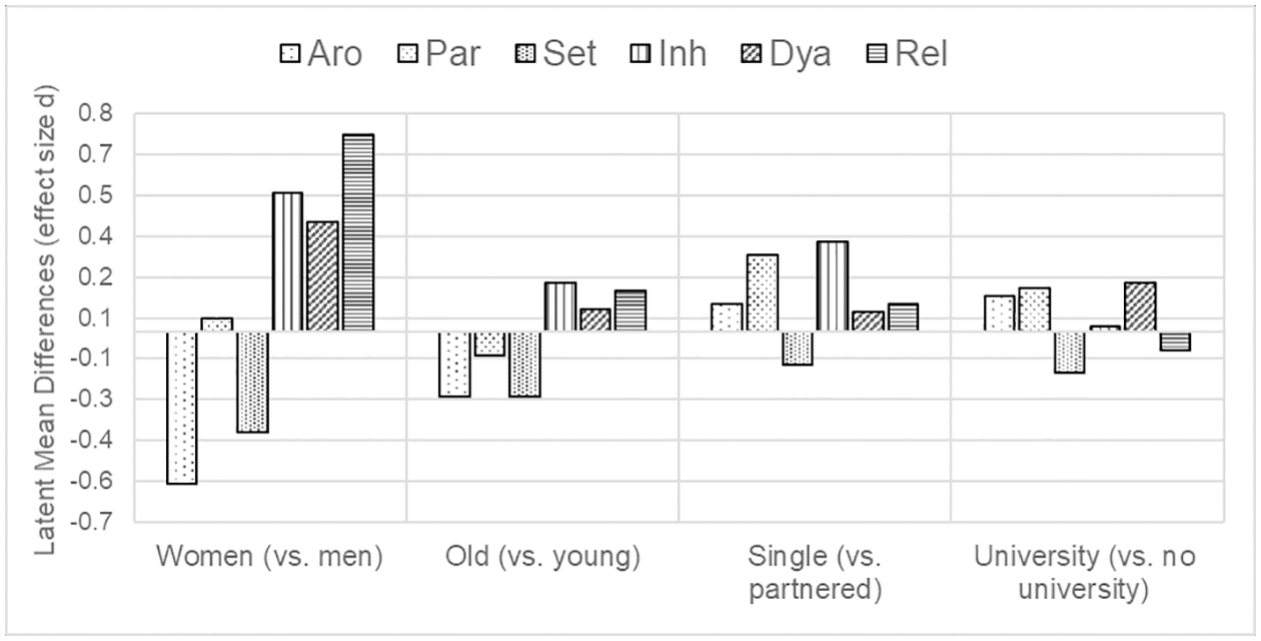
Latent mean differences of factors of the SESII-WM across different participant groups. Aro = Arousability (SE), Par = Partner characteristics and behaviors (SE), Set = Setting (SE), Inh = Inhibitory cognitions (SI), Dya = Dyadic elements of the sexual interaction (SI); Rel = Relationship importance (SI).

Measurement invariance testing revealed at least partial strong invariance across countries, genders, age groups, educational levels, and partnership status; thus, latent mean comparisons between all groups were feasible. To assess latent mean differences, the strong measurement invariant or partial strong measurement invariant models were used, respectively. Latent means were not compared across countries as the two samples differed significantly on several important variables such as age, relationship status, and education. Fig 1 shows the latent mean differences for the six SESII-W/M scales with one participant group fixed to zero.

Five out of six scales showed significant gender differences. Compared to men, women showed lower levels of SE (arousability, *z* = -13.97, *p* < .001, *d* = 0.56, and setting, *z* = -9.32, *p* < .001, *d* = 0.37) and higher levels of SI (relationship importance, *z* = 17.61, *p* < .001, *d* = 0.72, inhibitory cognitions, *z* = 12.82, *p* < .001, *d* = 0.51, dyadic elements of the sexual interaction, *z* = 10.03, *p* < .001, *d* = 0.40). Older participants scored lower on all three SE-factors (arousability: z = -6.10, p < .001, *d* = 0.24; partner behaviors and characteristics: *z* = -2.15, *p* = .032, *d* = 0.09; setting: *z* = -6.21, *p* < .001, *d* = 0.24), and higher on two SI-factors (inhibitory cognitions: *z* = 4.29, *p* < .001, *d* = 0.18; relationship importance: *z* = 4.45, *p* < .001, *d* = 0.15) than younger participants. Singles showed higher scores on the SE-scale partner behaviors and characteristics, *z* = 2.80, *p* = .005, *d* = 0.28, and higher levels of inhibitory cognitions (SI), *z* = 3.25, *p* = .001, *d* = 0.33, than partnered individuals. Participants with a university degree reported higher arousability (SE), *z* = 2.66, *p* = .008, *d* = 0.13, partner behaviors and characteristics (SE), *z* = 3.18, *p* = .001, *d* = 0.16, and dyadic interactions (SI), *z* = 3.36, *p* = .001, *d* = 0.18, and lower setting (SE), *z* = -3.13, *p* = .002, *d* = 0.15, than participants with a lower educational level.

#### SIS/SES-SF

**Fig 2:**
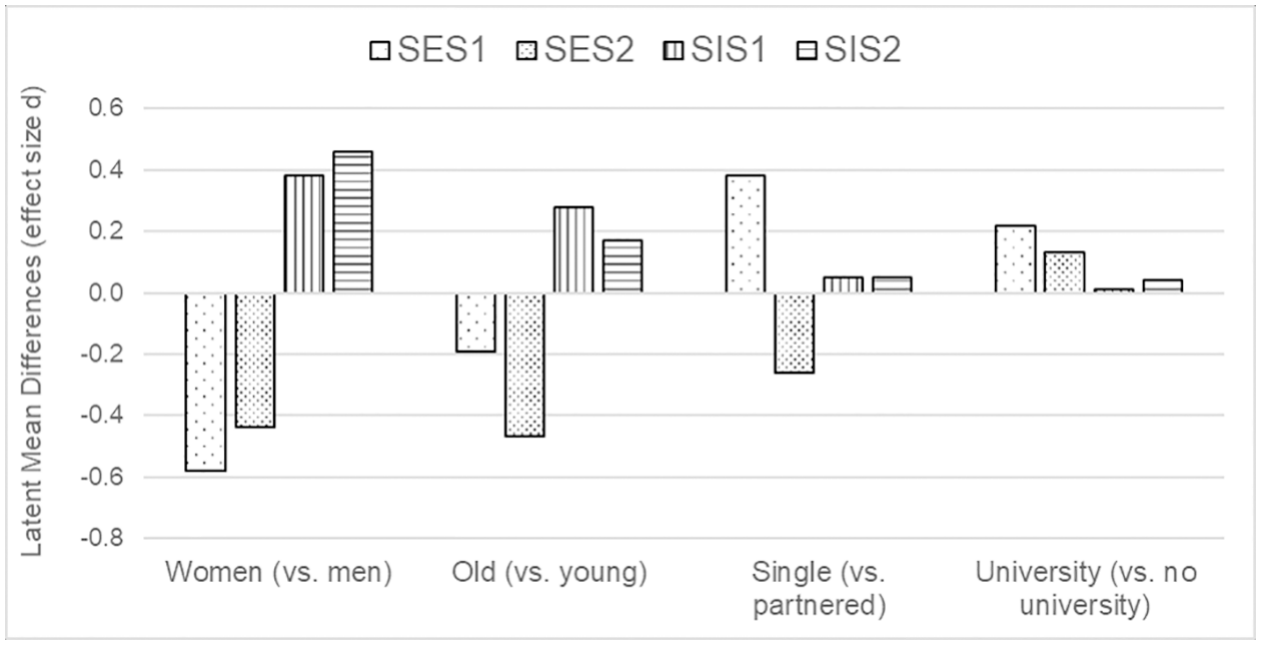
Latent mean differences in SIS/SES-SF factors across different participant groups.

Fig 2 shows the latent mean differences for the three SIS/SES-SF scales with one participant group fixed to zero.

Female participants reported lower SES1, *z* = -16.46, p < .001, *d* = 0.58, and SES2, *z* = -11.49, *p* < .001, *d* = 0.44, and higher SIS1, *z* = 10.22, *p* < .001, *d* = 0.38, and SIS2, *z* = 11.30, *p* < .001, *d* = 0.46, than male participants. The same pattern was found in older compared to younger participants (SES1: *z* = -5.60, *p* < .001, *d* = 0.19; SES2: z = -11.86, *p* < .001, *d* = 0.47; SIS1: *z* = 8.11, *p* < .001, *d* = 0.28; SIS2: *z* = 4.93, *p* < .001, *d* = 0.17). Participants who were single, reported higher SES1, *z* = 3.84, *p* < .001, *d* = 0.28, and lower SES2, *z* = -2.41, *p* = .016, *d* = 0.26, compared to participants in a steady relationship. Participants with university degree reported higher SES1, *z* = 4.79, *p* < .001, *d* = 0.22, and SES2, *z* = 2.70, *p* = .007, *d* = 0.13 than those without a university degree.

### Descriptive values

Descriptive values of both questionnaires are shown in Table 6.

**Table 6:** Description and internal consistency of the Sexual Excitation/Sexual Inhibition Inventory for Women and Men (SESII-WM) and the Sexual Inhibition Scales/Sexual Excitation Scales short form (SIS/SES-SF).

Scale (number of items)						
SESII-WM (mean scores)	*N*	*M*	*SD*	Skewness	Kurtosis	Cronbach’s alpha
Sexual excitation (14)	2607	2.38	0.42	0.10	0.33	.78
Arousability (5)	2482	2.60	0.55	-0.07	0.05	.72
Partner characteristics and behaviors (5)	2230	2.34	0.46	-0.11	0.29	.61
Setting (4)	2513	2.19	0.58	0.28	-0.15	.66
Sexual inhibition (16)	2570	2.64	0.47	-0.27	0.22	.85
Inhibitory cognitions (8)	2346	2.41	0.52	-0.08	0.01	.81
Dyadic elements of the sexual interaction (3)	2454	2.76	0.54	-0.39	0.51	.58
Relationship importance (5)	2341	2.73	0.59	-0.08	-0.23	.68
SIS/SES-SF (sum scores)						
SES (6)	2502	14.62	3.23	-.16	.16	.82
SIS1 (4)	2539	9.14	2.36	-.11	.42	.60
SIS2 (4)	2312	5.47	1.32	-.34	.04	.70

**Fig 3:**
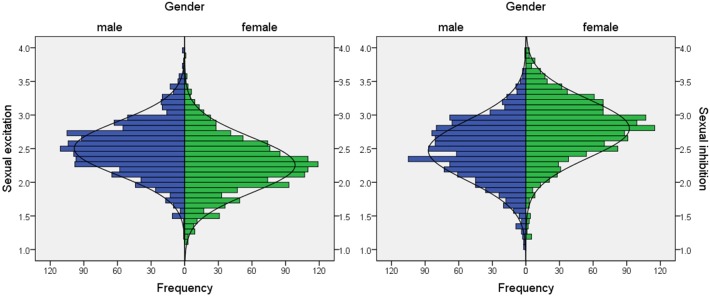
Distribution of the complete scales for SE (left) and SI (right) of the SESII-W/M in women and men.

**Fig 4:**
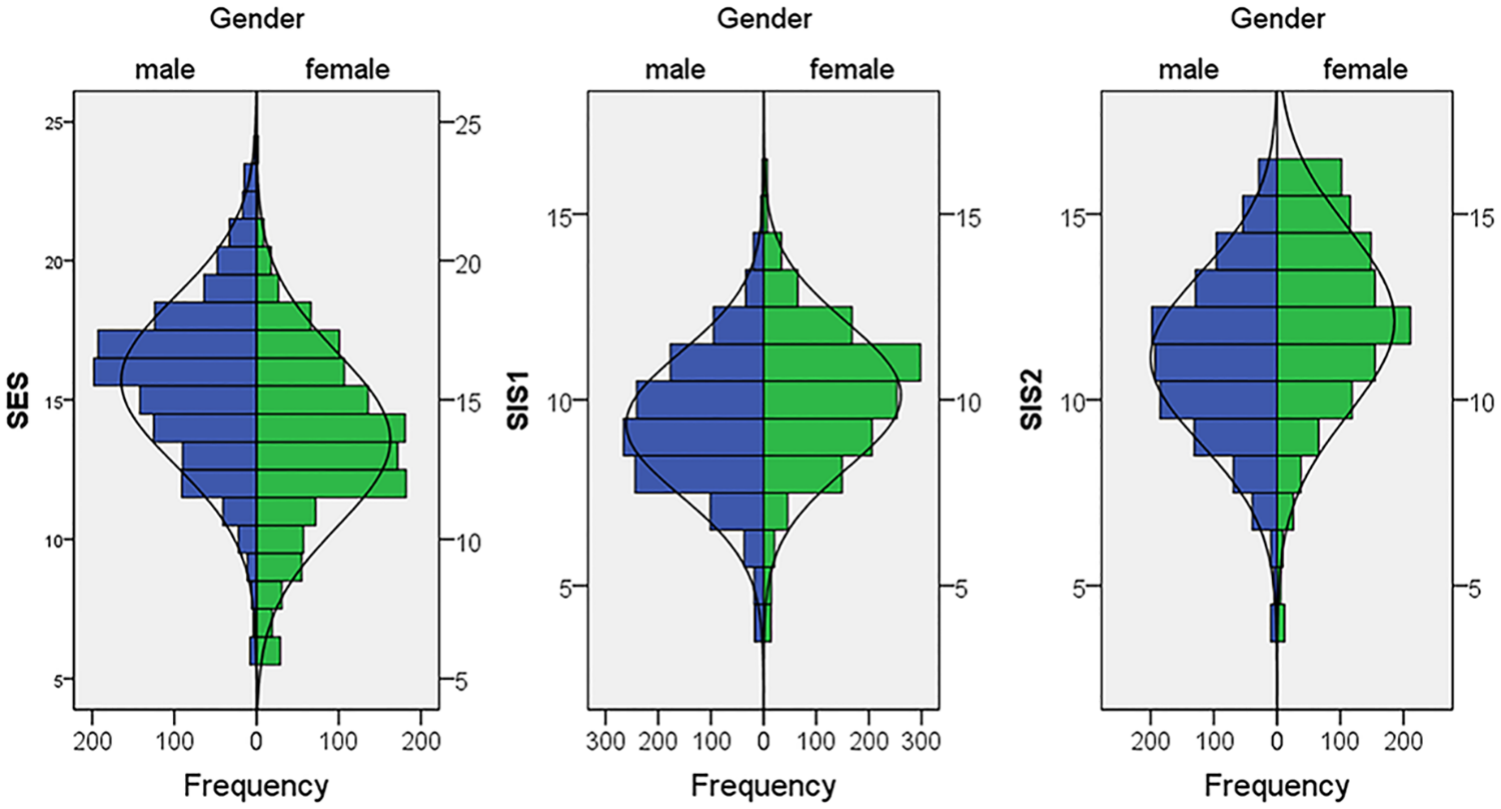
Distribution of the SES, SIS1, and SIS2 factors of the SIS/SES-SF in women and men.

See S1 Table for the descriptive values of the 24-item version of the SESII-W/M and the revised factors of the 4-factor 13-item version of the SIS/SES-SF. Skewness and kurtosis did not suggest a substantial deviation from non-normality. Distributions of the scales including all SE- and SI-related items of the SESII-W/M and the three scales of the SIS/SES-SF are shown in Figs 3 and 4.

#### Construct validity

Table 7 shows correlations for the scales of the original 30-item SESII-W/M, the original 14-item SIS/SES-SF and other distal and proximal variables.

**Table 7:** Bivariate correlations of the Sexual Excitation/Sexual Inhibition Inventory for Women and Men (SESII-WM) and the Sexual Inhibition Scales/Sexual Excitation Scales short form (SIS/SES-SF) with other proximal and distal variables.

Scale	Female sexual function (FSFI^1^)	Male sexual function (IIEF^2^)	Sociosexual orientation (SOI-R^3^)	Masturbation of frequency	Number of sexual partners	Satisfaction with life (SWLS^4^)	Anxiety, Depression (PHQ-4^5^)
SESII-WM							
Sexual excitation	.15**	.15**	.34**	.30**	.16**	-.02	.00
Arousability	.16**	.12**	.37**	.38**	.14**	-.02	.00
Partner characteristics and behaviors	.09**	.03	.13**	.12**	.06**	.00	.02
Setting	.10**	.18**	.26**	.18**	.14**	-.01	-.02
Sexual inhibition	-.17**	-.23**	-.30**	-.21**	-.14**	-.02	.10**
Inhibitory cognitions	-.27**	-.30**	-.16**	-.15**	-.09**	-.08**	.15**
Dyadic elements of the sexual interaction	-.13**	-.18**	-.16**	-.09**	-.06**	-.02	.09**
Relationship importance	-.04*	-.13**	-.42**	-.28**	-.20**	.05**	.03*
SIS/SES-SF							
SES	.10**	.06**	.42**	.39**	.16**	-.06**	.03*
SIS1	-.16**	-.21**	-.13**	-.12**	-.08**	-.04**	.10**
SIS2	-.08**	-.14**	-.19**	-.15**	-.10**	.01	.05**

** p < .01,

* p < .05

Note.

^1^Female Sexual Function Index [32],

^2^International Index of Erectile Function [34],

^3^Sociosexual Orientation Inventory-revised [19],

^4^Satisfaction with Life Scale [40],

^5^Patient Health Questionnaire-4 [43]

Most SE-scales showed positive, and most SI-scales showed negative correlations with sexual function in women and men. Effects were mostly small, while the inhibitory cognitions scale (SI) of the SESII-W/M showed a medium-sized negative correlation with men’s sexual function (*r* = -.30). A more casual sociosexual orientation was associated with lower SI and higher SE. The largest correlations were found between the SOI-R and SES/SES1 (*r* = .42/.41), relationship importance (SI; *r* = -.42), and arousability (SE; *r* = .37). A similar pattern was found concerning the frequency of masturbation which showed highest correlations with SES/SES2 (*r* = .39/.36), and arousability (SE; *r* = .38). For the number of lifetime sexual partners, a comparable pattern emerged. Effect sizes were, however, mostly small. Most scales showed no or negligible correlations with life satisfaction and/or symptoms of anxiety/depression. Small effects were found for different aspects of SI and anxiety/depression, with inhibitory cognitions (SI) showing the strongest relationship (*r* = .15).

### Reliability

#### SESII-W/M

Internal consistency of the complete SE and SI scales was acceptable to good (*α* = .78 for SE and *α* = .85 for SI). Four of the six factors (partner characteristics and behaviors, setting, dyadic elements of the sexual interaction, and relationship importance) showed poor internal consistency. However, some of these scales only included as few as three items, which may have contributed to this finding [58].

#### SIS/SES-SF

SES showed good (*α* = .80), SIS2 acceptable (*α* = .70), and the original SIS1 poor (*α* = .60) internal consistency. The two factors SES1 and SES2, found via exploratory factor analysis in this study, showed sufficient reliability (*α* = .81/.71). The modified SIS1—with only two instead of three items—still showed low internal consistency (*α* = .65). Table 6 shows internal consistency for both questionnaires.

## Discussion

The objective of this study was to evaluate the psychometric properties of the German SESII-W/M and SIS/SES-SF using a large population-based sample. The following sections will discuss our findings with respect to the factor structure, measurement invariance, latent mean differences, reliability, and construct validity of both questionnaires.

### Factor structure

In this study, we were unable to replicate the original factor solutions of the U.S. American scales without modifications. Fit indices used to assess model fit showed unacceptable values for the original 30-item version of the SESII-W/M and the 3-factor 14-item version of the SIS/SES-SF. Different solutions were found for improving model fit and for identifying a factor structure that fit our data. For the SESII-W/M, deleting as much as six ill-fitting items improved CFI and RMSEA enough to meet the requirements for a good model fit [46,47]. All six items showed significant factor loadings on their original factors, however, they also loaded on several other factors. Largest modification indices, indicating substantial double loadings, were found for Item 18 of the relationship importance factor (“If I am very sexually attracted to someone, I don’t need to be in a relationship with that person to become sexually aroused.”). This item showed significant factor loadings on five out of six factors of the SESII-W/M. This finding implies that this item (and other ill-fitting items) can be understood in different ways: While for some participants, agreeing to Item 18 would suggest low levels of SI, in the way that they don’t feel inhibited by a lack of commitment or trust, for others agreeing to this item can indicate a high general arousability (“I can get aroused easily, regardless of the situation”). Overall, our findings suggest that some items of the German SESII-W/M do not differentiate adequately between SE and SI.

With regards to the SIS/SES-SF, model fit of the original scale was unacceptable and did not improve by simply eliminating ill-fitting items. An exploratory factor-analysis revealed that a 4-factor solution fit out data best. Item 10 (“When I start fantasizing about sex, I quickly become sexually aroused.”) and Item 11 (“When I see others engaged in sexual activities, I feel like having sex myself.”) of the original SES factor constitute a second SE-factor in our sample. A potential explanation could be that some items related to SE were interpreted differently by our participants who were on average 30 years older than those who have partaken in the U.S. studies [16,24]. More than 70% of participants in our study were married, while approx. 90% of participants in the SESII-W/M validation study indicated being single/never married. These two sociodemographic variables alone should influence access to and evaluation of sexual stimuli (e.g., sexual partners, erotic videos) and may impact the factor structure of the SIS/SES-SF. A possible difference between SES1 and SES2 might also be that the former represents a more responsive sexual desire that is triggered by another person (i.e., an erotic voice, eye contact with an attractive person) [60], while the latter reflects more spontaneous sexual desire/excitability. For sexual fantasies (Item 10), the intrinsic quality of arousal is obvious, but viewing others during sexual activity (Item 11) also includes active initiative from the person as this might usually imply consuming pornographic material or seeking up situations in which sexual interactions of others can be observed.

To meet the requirements for an acceptable model fit, Item 9 of SIS1 (“Once I have an erection/am sexually aroused, I want to start intercourse right away before I lose my erection/arousal.”)—which also showed high factor loadings on SES2—was removed. A possible explanation for Item 9 showing loadings on factors of SE could be that some participants focused their attention on the first part of the item, which can be understood as an indication of high excitability and not wanting to delay sexual activity, without taking the second part (“…before I lose my erection/arousal”) into account.

The original 14-item SIS/SES-SF was based on the 45-items of the SIS/SES. In this study, however, only the short form was administered. To develop the most appropriate German short form of the SIS/SES, future studies may include the complete 45-item measure to identify which items constitute the best German short form.

### Measurement invariance

The 24-item SESII-W/M and the 4-factor 13-item SIS/SES-SF showed an acceptable to good model fit in the complete sample as well as in seven out of eight subgroups, namely men, younger and older participants, singles and persons in steady relationships, and individuals with and without university degree. For the 24-item SESII-W/M, the CFI did not quite meet the cut-off for an acceptable model fit in women. As the RMSEA suggested a good model fit and a CFI of .876 was comparable to those reported in the original validation study of the U.S. scale [24], we decided to proceed with measurement invariance testing. However, this finding is in line with previous studies on SE and SI that reported a slightly worse model fit for the SIS/SES [12] and SESII-W/M [24] in female participants. Both questionnaires under investigation exhibited strong measurement invariance for singles and partnered individuals, older and younger persons, as well as for participants with and without university degree. The SIS-SES/SF was also strong measurement invariant across genders.

Thresholds of Item 1 (“Sometimes I have so many worries that I am unable to get aroused.”) and Item 9 (“I think about sex a lot when I am bored.”) were freed to achieve partial scalar measurement invariance for the SESII-W/M across genders. Thresholds of Item 1 were higher and thresholds of Item 9 were lower in male compared to female participants. This means that, given a certain level of the underlying traits—inhibitory cognitions for Item 1 and arousability for Item 9—men were less likely to endorse Item 1 and more likely to endorse Item 9 than women (i.e., less/more of the underlying trait was needed for men to endorse the items).

Partial strong measurement invariance was also established for both questionnaires across German and U.S. American versions of the questionnaires. For the SIS/SES-SF, Item 5 (“If I am masturbating on my own and realize that someone is likely to come into the room at any moment, I will lose my erection/my sexual arousal.”) was a source of invariance, with the German sample showing higher item thresholds than the U.S. sample. Given a certain level of SIS2, German participants were less likely to endorse Item 5 than U.S. participants.

For the SESII-W/M, thresholds of Item 3 (“When I think about someone I find sexually attractive, I easily become sexually aroused.”) and Item 10 (“I find it arousing when a partner does something nice for me.”) were higher in the U.S. than in the German sample. This means that, given a certain level of the underlying traits—arousability for Item 3 and partner characteristics and behaviors for Item 10—American participants were less likely to endorse these items than German participants. While these findings could implicate potential cultural differences or translation issues, both samples differed in important other variables such as age, relationship status, and education which could very well contribute to this invariance. With the exception of these items, the statistical requirements for comparisons of the relationships between latent variables as well as latent mean comparisons across groups were fulfilled.

### Latent mean differences

As both instruments were at least partial scalar measurement invariant across all participant groups under investigation, latent mean comparisons were allowed based on the 24-item version of the SESII-W/M and the 4-factor 13-item version of the SIS/SES-SF across all subsamples. Largest between-group differences were found between men and women. Across both questionnaires and all factors, men showed significantly higher SE, and lower SI than women. Largest gender-differences were found concerning relationship importance (*d* = 0.72), SES1 (*d* = 0.58), arousability (*d* = 0.56), inhibitory cognitions (*d* = 0.51), SIS2 (*d* = 0.46), SES2 (*d* = 0.44), and SIS1 (*d* = 0.38). This finding is in line with previous studies that reported such gender-differences based on observed scores, not latent means [12,21,24]. These results are also in accordance with the dual control model’s assumptions [2] and can be explained by evolutionary mechanisms [61] as well as differences in social learning [62].

Partnership status was also associated with smaller, but still significant latent mean differences. Single individuals reported being more easily aroused by partner characteristics and behaviors than participants who were in a committed partnership (*d* = 0.28). A possible explanation for this finding could be that different frames of reference were used by participants with and without a steady partner: While singles imagined how arousing they would find certain partner variables in theory, partnered individuals might think about their current partner, recent situations (i.e., a partner doing chores) and their specific reactions to this behavior (i.e., not being aroused). In addition, singles reported higher levels of inhibitory cognitions (*d* = 0.33). While some singles are not sexually active, others are having sex with casual acquaintances or are on the search for a new partner [63]. Except for extra-relationship affairs or newly started partnerships, the likelihood of having sex with a relatively new partner with unfamiliar sexual preferences or practices should be higher for singles compared to partnered individuals. Having sex with someone new may activate cognitions of self-doubt (i.e., Am I a good enough lover?), shyness or self-consciousness. Also, with a new or casual partner, one may be reluctant to communicate sexual wishes or needs and may therefore experience more inhibitory cognitions related to one’s own ability to get aroused or perform sexually.

Older participants showed significantly lower SE-, and higher SI-scores across both questionnaires. Largest age-differences were found concerning SES2 (*d* = 0.47), which includes statements about arousability by sexual phantasies and by seeing others engaging in sex. This finding is in line with studies suggesting a decline in sexual desire with age [64–66]. As the likelihood for sexual dysfunctions such as erectile problems or low desire increases with age [67], concerns or worries about sexual performance may become more salient. The age-related pattern is in line with the one other study that used the SIS/SES-SF in a population-based representative sample [14,21]. Their analysis, however, revealed an interaction between the effect of age and gender on SIS1, with men showing a linear increase in SIS1 with age, and women showing a u-shaped pattern with highest values reported between 40 and 50 years of age. Using a dichotomous age-variable prevented us from identifying more subtle age-dependent effects. As another limitation, our study design does allow us to disentangle cohort effects from age effects. To close this gap in the literature, longitudinal studies are recommended.

Educational level—operationalized as having vs. not having a university degree—was associated with higher SE concerning SES1 (*d* = 0.22), partner characteristics and behaviors (*d* = .16), arousability (*d* = 0.13), and SES2 (*d* = 0.13). Increased levels of SE for highly educated people have also been reported in a previous representative study using the SIS/SES-SF [14]. Future studies might clarify, whether differences in socialization (i.e., a more liberal upbringing) or mediating variables such as religiosity or (mental) health might explain these group differences. Effects were, however, mostly negligible to small. Participants with university degree reported higher inhibition due to dyadic aspects of sexual interactions (*d* = 0.18), suggesting that they require a balance in giving and/or receiving pleasure and affirmation about a partner’s feelings in order to get aroused. A potential implication of this finding may be that in more highly educated participants, traditional sex role attitudes concerning the man being the more active sexual partner play less of a role [68,69].

### Reliability

Internal consistency was calculated as a measure of reliability. While internal consistency of the two complete scales of SE and SI of the SESII-W/M, as well as SES, SIS2, SES1 (original scales), and SES2 (new scale) of the SIS/SES-SF was acceptable to good, other scales did not meet the cut-off for at least acceptable consistency. Low homogeneity was, for instance, found for the dyadic elements of the sexual interaction factor of the SESII-W/M. This 3-item factor describes how different interpersonal aspects reduce or inhibit arousal. While two items focus on aspects of the sexual interaction itself (i.e., balance of giving and receiving pleasure, having a partner who is sensitive to sexual signals), the remaining item describes how being insecure about a partner’s feeling towards the relationship impacts sexual response. In addition to the unsatisfactory internal consistency, this scale also showed modest factor loadings in the CFA. Taken together, the authors interpret these findings in that this scale may not reflect a single underlying dimension, but rather different aspects of SI. Another inconsistent scale is SIS1 of the SIS/SES-SF. Excluding one of the four items of this scale—namely, Item 9 which was also found problematic in our CFA and was deleted from the final factor solution—improved internal consistency from α = .60 to α = .65. While some of the shorter scales fail to meet the cut-off for acceptable homogeneity in our study, results are comparable to previous studies investigating the reliability of other original and translated SE- and SI-measures [4,24,29,70]. To overcome another limitation of this study, future studies should include an evaluation of the test-retest reliability of the German SESII-W/M and SIS/SES-SF.

### Construct validity

SE and SI correlated with other sexual behaviors or constructs in the expected directions. In line with the theoretic assumptions of the dual control model [1,2] and previous research [4–6], higher levels of SE and lower levels of SI were associated with higher sexual function in both genders. Inhibitory cognitions (SI) showed the strongest, medium-sized negative association with sexual function in both women and men. Correlations between the factors of the dual control model and sexual function were similar in both genders. While one study has found evidence for SI being a risk factor for future sexual dysfunction [5], more research is needed to evaluate the direction of effects. Sociosexual orientation, which describes attitudes and behaviors related to sex outside of committed relationships showed positive associations with SE and negative with SI. Individuals who reported not needing trust and commitment in order to get aroused (relationship importance scale of SESII-W/M) and are easily aroused by a variety of stimuli (arousability scale of SESII-W/M, SES scale of SIS/SES-SF) reported a more causal sociosexual orientation. The pattern of results was similar with respect to frequency of masturbation and number of sexual partners. Differences were found concerning the size of the effects: While attitudes towards casual sex and masturbation showed small to medium correlations, the associations with the number of partners was somewhat smaller. While the frequency of masturbation may be more closely linked to sexual desire, the number of partners may also be influenced by external factors such as access to sexual partners, physical attractivity, or status [71,72]. To investigate if SE and SI are related to an evaluation of life in general (i.e., being more satisfied with life may lead participants to answer items in a more positive or affirmative way) associations with symptoms of anxiety/depression and life satisfaction were assessed. While associations with life satisfaction were negligible, some aspects of SI (inhibitory cognitions, dyadic elements of a sexual interaction, SIS1) showed small, positive correlations with anxious/depressive symptoms. These findings can be interpreted in the light of the substantial associations between sexual dysfunctions and depression [73]. However, in which way these three variables—sexual dysfunction, depression, and SI—influence each other cannot be clarified with this study. Some of the questionnaires that have been regularly used to assess the construct validity of instruments assessing SE and SI such as the Behavioral Activation Scales/Behavioral Inhibition Scales [17] or the Sexual Sensation Seeking Scale [25] were not administered in the present study. A previous study assessing the psychometric properties of the SESII-W—that includes 19 items which are also part of the SESII-W/M—found correlations with these scales in the expected size and direction [29]. Combined with the pattern of correlations found in the present study, we summarize the construct validity of the instruments under investigation as sufficient.

### Limitations

Some limitations threaten the internal and external validity and reliability of our findings. The volunteer bias that is known in sex-research may have been relevant for our study [74]. Individuals with more conservative attitudes may have felt uncomfortable with the study topic and have been unlikely to participate. Thus, the sample might not be fully representative for the German adult population. During the initial telephone screening, individuals in steady relationships were encouraged to motivate their partner to participate as well. Couples with relationship discord are therefore most likely underrepresented in our study. Some questionnaires that are commonly used to assess the construct validity of instruments measuring SE and SI such as the Behavioral Activation Scales/Behavioral Inhibition Scales [17] were not used in this study. Therefore, convergent and discriminant validity could not be examined as thoroughly as it had been in other studies [4,11]. In addition, test-retest reliability of the scales was not examined. While CFA is commonly used to confirm the factor structure of multidimensional psychometric instruments [52,53], it has been criticized for being overly restrictive in its assumption that each item loads on one factor only [75]. Thus, the less than sufficient model fit that was found in some of our analyses is potentially associated to our reliance on CFA. Using alternative techniques such as exploratory structural equation modeling (ESEM) might be beneficial to fully evaluate the factor structure of SESII-W/M and SIS/SES-SF [75].

### Conclusion

Our study is the first to evaluate the psychometric properties of the German SESII-W/M and SIS/SES-SF in a large population-based sample. Our findings indicate that both questionnaire can be used to assess SE and SI in German-speaking samples. When using our scale to compare different participant groups, researchers should consider using our revised versions of both questionnaires as the original factor structures did not fit our representative population data. More research is needed to investigate whether the 24-item SESII-W/M and the 13-item 4-factor SIS/SES-SF can be replicated in other samples. The SIS/SES-SF can be recommended for research settings where time is limited. To address specific research questions—for example how different aspects of SI impact erectile function in men or how arousability by partner characteristics can influence the likelihood of cheating on a committed partner—the SESII-W/M may be more suitable.

## Supporting information

S1 Table Description and reliability of the revised scales of SESII-WM and SIS/SES-SF. (DOCX)

S1 File Dataset. (SAV)

S2 File Paper-pencil version of the German SESII-W/M. (DOCX)
