# Supplementary material for: Psychometric properties of the Sexual Excitation/Sexual Inhibition Inventory for Women and Men (SESII-W/M) and the Sexual Excitation Scales/Sexual Inhibition Scales short form (SIS/SES-SF) in a population-based sample in Germany
Source: PLoS One. 2018 Mar 12;13(3):e0193080. doi: 10.1371/journal.pone.0193080 (PMC5846736; doi:10.1371/journal.pone.0193080)
Supplement: S1 Table — (DOCX) [file pone.0193080.s001.docx]

**S X. Table.** **Description and reliability of the revised scales of the Sexual Excitation/Sexual Inhibition Inventory for Women and Men (SESII-WM) and the Sexual Inhibition Scales/Sexual Excitation Scales short form (SIS/SES-SF).**

| Revised scale* (number of items) | | | | | | | |
| --- | --- | --- | --- | --- | --- | --- | --- |
| SESII-WM (mean scores) | *N* | *M* | *SD* | Skewness | Kurtosis | | Cronbach’s alpha |
| Sexual excitation (11) | 2630 | 2.32 | 0.45 | .06 | .17 | | .70 |
| Partner characteristics and behaviors (4) | 2278 | 2.29 | 0.47 | -.05 | .33 | | .56 |
| Setting (2) | 2591 | 2.10 | 0.77 | .29 | -.63 | | .69 |
| Sexual inhibition (16) | 2580 | 2.67 | 0.49 | -.29 | .29 | | .84 |
| Inhibitory cognitions (6) | 2379 | 2.39 | 0.54 | -.05 | -.07 | | .76 |
| Relationship importance (4) | 2377 | 2.83 | 0.64 | -.19 | -.29 | | .67 |
| SIS/SES-SF (sum scores) | | | | | | | |
| SES1 (4) | 2544 | 9.14 | 2.36 | -0.07 | -0.14 | .81 | |
| SES2 (2) | 2596 | 5.47 | 1.32 | -0.34 | 0.24 | .71 | |
| SIS1 (3) | 2553 | 7.47 | 1.70 | -0.12 | 0.17 | .65 | |
| Note. * Unrevised scales (Arousability, Dyadic elements of the sexual interaction, and SIS2) are shown in Table 6. | | | | | | | |
