# Supplementary material for: Psychometric properties of the Sexual Excitation/Sexual Inhibition Inventory for Women and Men (SESII-W/M) and the Sexual Excitation Scales/Sexual Inhibition Scales short form (SIS/SES-SF) in a population-based sample in Germany
Source: PLoS One. 2018 Mar 12;13(3):e0193080. doi: 10.1371/journal.pone.0193080 (PMC5846736; doi:10.1371/journal.pone.0193080)
Supplement: S2 File — (DOCX) [file pone.0193080.s003.docx]

**Sexual Excitation/Sexual Inhibition Inventory for Women and Men**

Deutsche Version nach Velten, Scholten und Margraf (2017)

| Sie lesen nun einige Aussagen über Aspekte oder Situationen, die Ihre sexuelle Erregung beeinflussen können.  Sexuelle Erregung wird dabei beschrieben als anmachen, an- oder abtörnen oder erregt sein. Menschen erleben sexuelle Erregung ganz unterschiedlich. Sie nehmen sie im Genitalbereich (z.B. feucht oder hart werden, kribbeln oder Wärme), an anderen Körperregionen (z. B. Herzklopfen, Temperaturänderungen oder Hautempfindungen) oder als Gefühle (z. B. Vorfreude oder sich sexy fühlen) wahr.  Wir möchten wissen, wie Sie persönlich *am* *wahrscheinlichsten* auf die geschilderten Situationen reagieren. Manche Aussagen treffen vielleicht nicht auf Ihre aktuelle Situation zu. Bitte denken Sie dann daran, wie Sie wahrscheinlich in einer solchen Situation reagieren würden.  Bitte lesen Sie jede Aussage sorgfältig und markieren Sie die zu Ihnen passende Antwort. Auch wenn einige Feststellungen ähnlich klingen, sind doch alle etwas anders. Denken Sie bitte nicht zu lange nach und versuchen Sie spontan und ehrlich zu antworten.  *Anmerkung: Mit dem Begriff Partner sind auch Partner*innen gemeint.* |
| --- |

|  |  | Stark ablehnen  1 | Ablehnen  2 | Zustimmen  3 | Stark zustimmen  4 |
| --- | --- | --- | --- | --- | --- |
| 1. | Manchmal habe ich so viele Sorgen, dass ich nicht in der Lage bin, erregt zu werden. | 1 | 2 | 3 | 4 |
| 2. | Wenn ich denke, dass ich sexuell benutzt werde, törnt mich das völlig ab. | 1 | 2 | 3 | 4 |
| 3. | Wenn ich an jemanden denke, den ich sexuell attraktiv finde, fällt es mir leicht, sexuell erregt zu werden. | 1 | 2 | 3 | 4 |
| 4. | Einen Partner dabei zu sehen, wie er/sie sein/ihr Talent unter Beweis stellt, kann mich sexuell sehr erregen. | 1 | 2 | 3 | 4 |
| 5. | Wenn die Möglichkeit besteht, dass uns jemand beim Sex sehen oder hören könnte, ist es schwieriger für mich, erregt zu werden. | 1 | 2 | 3 | 4 |
| 6. | Wenn ich unsicher bin, was ein Partner für mich empfindet, ist es schwieriger für mich, erregt zu werden. | 1 | 2 | 3 | 4 |
| 7. | Wenn ich spüre, dass eine sexuelle Reaktion von mir erwartet wird, habe ich Schwierigkeiten, erregt zu werden. | 1 | 2 | 3 | 4 |
| 8. | Wenn jemand etwas tut, was seine Intelligenz zeigt, törnt mich das an. | 1 | 2 | 3 | 4 |
| 9. | Ich denke viel an Sex, wenn mir langweilig ist. | 1 | 2 | 3 | 4 |
| 10. | Ich finde es erregend, wenn ein Partner etwas Nettes für mich tut. | 1 | 2 | 3 | 4 |
| 11. | Manchmal fühle ich mich beim Sex so schüchtern und unsicher, dass ich nicht vollständig erregt werden kann. | 1 | 2 | 3 | 4 |
| 12. | Allein über Sex zu reden genügt, um mich in sexuelle Stimmung zu versetzen. | 1 | 2 | 3 | 4 |
| 13. | Beim Sex verringert es wirklich meine Erregung, wenn mein Partner nicht feinfühlig auf die Signale, die ich gebe, reagiert. | 1 | 2 | 3 | 4 |
| 14. | Ich finde es schwieriger sexuell erregt zu werden, wenn andere Menschen in der Nähe sind. | 1 | 2 | 3 | 4 |
| 15. | Wenn ich darüber nachdenke, ob ich zum Orgasmus komme, ist es für mich viel schwieriger, erregt zu werden. | 1 | 2 | 3 | 4 |
| 16. | Es wäre schwierig für mich, bei jemandem sexuell erregt zu werden, der mit einer anderen Person eine Beziehung oder ein sexuelles Verhältnis hat. | 1 | 2 | 3 | 4 |
| 17. | Manchmal fühle ich mich zu jemandem so hingezogen, dass ich nicht verhindern kann, sexuell erregt zu werden. | 1 | 2 | 3 | 4 |
| 18. | Wenn mich jemand sexuell stark anzieht, brauche ich nicht in einer Beziehung mit der Person sein, um sexuell erregt zu werden. | 1 | 2 | 3 | 4 |
| 19. | Es ist für mich schwierig, erregt zu werden, wenn nicht „alles richtig“ ist. | 1 | 2 | 3 | 4 |
| 20. | Es beeinträchtigt meine Erregung, wenn es beim Sex kein Gleichgewicht zwischen Genussbereiten und -empfangen gibt. | 1 | 2 | 3 | 4 |
| 21. | Es törnt mich wirklich an, wenn ich daran denke, dass ich beim Sex erwischt werden könnte. | 1 | 2 | 3 | 4 |
| 22. | Wenn ich mir Sorgen darüber mache, dass ich zu lange brauche, um erregt zu werden, kann das meine Erregung beinträchtigen. | 1 | 2 | 3 | 4 |
| 23. | Wenn ich sehe, dass ein Partner gut mit anderen auskommt, werde ich leichter sexuell erregt. | 1 | 2 | 3 | 4 |
| 24. | Einem Partner körperlich nah zu sein genügt bereits, um mich anzutörnen. | 1 | 2 | 3 | 4 |
| 25. | In einer anderen Umgebung als gewöhnlich Sex zu haben, törnt mich richtig an. | 1 | 2 | 3 | 4 |
| 26. | Beim Sex muss ich mich auf meine eigenen sexuellen Gefühle konzentrieren, um erregt zu bleiben. | 1 | 2 | 3 | 4 |
| 27. | Wenn ich denke, dass ein Partner mich emotional verletzen könnte, blocke ich sexuell ab. | 1 | 2 | 3 | 4 |
| 28. | Ich muss einem Partner wirklich vertrauen, um sexuell vollkommen erregt zu werden. | 1 | 2 | 3 | 4 |
| 29. | Wenn ich mir Sorgen darüber mache, ob ich ein/e gute/r Liebhaber/in bin, ist es unwahrscheinlicher, dass ich erregt werde. | 1 | 2 | 3 | 4 |
| 30. | Wenn ein Partner mich überrascht indem er/sie den Haushalt macht, entfacht das mein sexuelles Interesse. | 1 | 2 | 3 | 4 |

**Scoring der originalen 30-Item Version:**

| Skala | Mittelwerte der Items |
| --- | --- |
| Sexuelle Excitation (engl. sexual excitation) | 3, 4, 5*, 8, 9, 10, 12, 14*, 17, 21, 23, 24, 25, 30 |
| Sexuelle Inhibition (engl. sexual inhibition) | 1, 2, 6, 7, 11, 13, 15, 16, 18*,19, 20, 22, 26, 27, 28, 29 |
| Erregbarkeit (engl. Arousability) | 3, 9, 12, 17, 24 |
| Partnereigenschaften und -verhalten (engl. Partner characteristics and behavior) | 4, 8, 10, 23, 30 |
| Umgebung (engl. setting) | 5*, 14*, 21, 25 |
| Hemmende Kognitionen (engl. inhibitory cognitions) | 1, 7, 11, 15, 19, 22, 26, 29 |
| Beziehungswichtigkeit (engl. relationship importance) | 2, 16, 18*, 27, 28 |
| Dyadische Elemente der sexuellen Interaktion (engl. dyadic elements of the sexual interaction) | 6, 13, 20 |

*umgekehrt kodiert

**Scoring der revidierten deutschen 24-Item Version:**

| Skala | Mittelwerte der Items |
| --- | --- |
| Sexuelle Excitation (engl. sexual excitation) | 3, 5*, 8, 9, 10, 12, 14*, 17, 23, 24, 30 |
| Sexuelle Inhibition (engl. sexual inhibition) | 1, 2, 6, 7, 11, 13, 15, 16, 20, 26, 27, 28, 29 |
| Erregbarkeit (engl. Arousability) | 3, 9, 12, 17, 24 |
| Partnereigenschaften und -verhalten (engl. Partner characteristics and behavior) | 8, 10, 23, 30 |
| Umgebung (engl. setting) | 5*, 14* |
| Hemmende Kognitionen (engl. inhibitory cognitions) | 1, 7, 11, 15, 26, 29 |
| Beziehungswichtigkeit (engl. relationship importance) | 2, 16, 27, 28 |
| Dyadische Elemente der sexuellen Interaktion (engl. dyadic elements of the sexual interaction) | 6, 13, 20 |

*umgekehrt kodiert

**Quellangaben:**

Milhausen, R. R., Graham, C. A., Sanders, S. A., Yarber, W. L., Maitland, S. B., Yarber, W. L., & Maitland, S. B. (2010). Validation of the Sexual Excitation / Sexual Inhibition Inventory for Women and Men. Archives of Sexual Behavior, 39(5), 1091–1104. https://doi.org/10.1007/s10508-009-9554-y

Velten, J., Scholten, S., & Margraf, J. (2017). Psychometric properties of the Sexual Excitation/Sexual Inhibition Inventory for Women and Men (SESII-W/M) and the Sexual Excitation Scales/Sexual Inhibition Scales short form (SIS/SES-SF) in a population-based sample in Germany. PloS One.
